# Rapid preparation of mutated influenza hemagglutinins for influenza virus pandemic prevention

**DOI:** 10.1186/s13568-016-0179-y

**Published:** 2016-01-21

**Authors:** Ryosuke Nishioka, Atsushi Satomura, Junki Yamada, Kouichi Kuroda, Mitsuyoshi Ueda

**Affiliations:** Division of Applied Life Sciences, Graduate School of Agriculture, Kyoto University, Sakyo-Ku, Kyoto, 606-8502 Japan; Japan Society for the Promotion of Science, Sakyo-Ku, Kyoto, 606-8502 Japan

**Keywords:** Influenza, Hemagglutinin, Yeast display, Hemagglutination assay

## Abstract

Influenza viruses have periodically caused pandemic due to frequent mutation of viral proteins. Influenza viruses have two major membrane glycoproteins: hemagglutinin (HA) and neuraminidase (NA). Hemagglutinin plays a crucial role in viral entry, while NA is involved in the process of a viral escape. In terms of developing antiviral drugs, HA is a more important target than NA in the prevention of pandemic, since HA is likely to change the host specificity of a virus by acquiring mutations, thereby to increase the risk of pandemic. To characterize mutated HA functions, current approaches require immobilization of purified HA on plastic wells and carriers. These troublesome methods make it difficult to respond to emerging mutations. In order to address this problem, a yeast cell surface engineering approach was investigated. Using this technology, human HAs derived from various H1N1 subtypes were successfully and rapidly displayed on the yeast cell surface. The yeast-displayed HAs exhibited similar abilities to native influenza virus HAs. Using this system, human HAs with 190E and 225G mutations were shown to exhibit altered recognition specificities from human to avian erythrocytes. This system furthermore allowed direct measurement of HA binding abilities without protein purification and immobilization. Coupled with the ease of genetic manipulation, this system allows the simple and comprehensive construction of mutant protein libraries on yeast cell surface, thereby contributing to influenza virus pandemic prevention.

**Electronic supplementary material:**

The online version of this article (doi:10.1186/s13568-016-0179-y) contains supplementary material, which is available to authorized users.

## Introduction

Influenza viruses have caused pandemic in humans as well as in animals (Potter 2001; Capua and Alexander 2008). The human influenza viruses recognize human-specific cell receptors via α2,6-linked sialic acid side chains on surface glycoproteins, while avian influenza viruses recognize avian-specific cell receptors via α2,3-linked sialic acid side chains on surface glycoproteins (Gagneux et al. 2003; Ito and Kawaoka 2000). These subtle differences in sialic acid recognition are key in preventing animal influenza viruses from infecting humans; however, some influenza viruses infecting animals have acquired the potency to overcome this difference (Van Reeth 2007). Highly pathogenic avian influenza viruses including H5N1 subtypes have been reported to alter their targets from avian to human, causing 447 deaths until 2010 (Webby and Webster 2003; WHO 2015). Recently, the swine influenza virus also acquired the ability to infect humans via genetic re-assortment (Zhou et al. 1999). The swine influenza virus was transmitted widely among humans, resulting in the first influenza pandemic in the twenty first century (Kuiken et al. 2011). Swine may play roles as intermediate hosts for influenza viruses between humans and avian species because swine cell receptors have both the α2,6- and the α2,3-linked sialic acid side chains on their surface glycoproteins (Ito and Kawaoka 2000). When human influenza viruses and avian influenza viruses infect the same intermediate host, gene re-arrangement between the human and avian influenza viruses is likely to occur, enabling the viruses to alter their recognition preferences of sialic acid on surface glycoprotein (Dawood et al. 2009). Indeed, the influenza virus pandemic is thought to have emerged from gene re-assortment of the North American H3N2 human-like viruses and the H1N2 swine viruses with Eurasian avian-like swine viruses (Dawood et al. 2009). The re-assortment process enables animal influenza viruses to effectively develop the capacity to infect humans (Zhou et al. 1999), which suggests that the threat of infection caused by new influenza viruses will continue as the differences between human and animal viruses are overcome.

Influenza viruses have two major surface membrane glycoproteins: neuraminidase (NA) and hemagglutinin (HA) (Air and Laver 1989; Skehel and Wiley 2000). While NA is involved in the process by which newly generated viruses escape from host cells (Air and Laver 1989), HA, which forms a trimer embedded on the viral envelope surface, is responsible for binding and entry into host cells (Skehel and Wiley 2000). Each monomer of HA is synthesized as a single polypeptide (HA0) that is cleaved by host proteases into two subunits (HA1 and HA2) (Skehel and Wiley 2000), of which HA1 represents an active head region that binds to sialic acid side chains on surface glycoproteins and HA2 represents a stem region that is required for membrane fusion activity and infectivity (Skehel and Wiley 2000). Both NA and HA are frequently mutated in the life cycle of the influenza virus, resulting in high levels of antigenic variation (Domingo and Holland 1997).

The high mutation rate of HA sometimes generates new HA subtypes that have never infected humans. Some mutations have already been reported to contribute to the changes in the binding abilities of HA from avian to human receptors or from human to avian receptors, which were, in part, responsible for the pandemic (Stevens et al. 2006). Inhibition of HA prevents the infection as a first attack caused by influenza virus mutants. Various methods including phage display have been used to screen for inhibitors that bind to the target domains (Hawkins et al. 1992; Matsubara et al. 2010; Krag et al. 2006); however, these current approaches would not be sufficient to deal with influenza viruses for the following reasons. Firstly, in order to discriminate the binding abilities of HA mutants, purified HA needs to be immobilized on plastic wells or carriers (Hawkins et al. 1992; Matsubara et al. 2010). Secondly, frequent mutations make it difficult to deal with HA mutants because time-consuming methods are required for each mutant preparation. In order to address these problems, a novel method for dealing with the high mutation rate of the influenza virus must be developed.

We previously reported on the successful display of various head domains of the wild-type and drug-resistant NAs from the avian H5N1 virus on yeast cell surface using yeast cell surface engineering in *Saccharomyces cerevisiae* (Shigemori et al. 2013). In this system, a protein of interest can be displayed on yeast cell surface as a fusion protein with α-agglutinin, which is a cell wall protein involved in mating (Murai et al. 1997; Ueda and Tanaka 2000). This method allows direct measurement of the protein activity without protein purification steps (Ueda and Tanaka 2000).

Here, we report on the construction of two kinds of yeasts displaying either human short HA (hSHA^α2,6^) or human long HA (hLHA^α2,6^) from the human H1N1 virus. hSHA^α2,6^ consists of the domain that binds to α2,6-linked sialic acid side chains, while hLHA^α2,6^ consists of the binding domain and the stem region without the secretion signal peptide and the transmembrane domain. hSHA^α2,6^ and hLHA^α2,6^ displayed on the yeast cell surface were shown to recognize α2,6-linked sialic acid side chains on the surface glycoproteins of erythrocytes. The cell surface display of human HA mutants with altered binding specificity by induction of mutations was furthermore shown to result in direct detection of recognizing α2,3-linked sialic acid side chains on chicken erythrocytes. The system reported on here was shown to rapidly prepare HA mutants on cell surface and to allow for easy analysis of the functions of these mutants coupled with easy genetic manipulation of yeast. Our yeast cell surface system therefore allows for the simple and comprehensive construction of HA and NA mutant libraries on yeast cell surface, thereby facilitating the prevention of pandemic caused by influenza viruses.

## Materials and methods

### Microbial strains and culture media

The DH5α (F^−^, *end*A1, *hsd*R17[r_k_^−^/m_K_^+^], *sup*E44, *thi*-1, *λ*^−^, *deo*R, *rec*A1, *gyr*A96, *pho*A, φ80d*lac*ZΔM15, Δ[*lac*ZYA-*arg*F]U169) *Escherichia coli* strain (Toyobo, Osaka, Japan) was used as the host for recombinant DNA manipulation. *S. cerevisiae* BY4741/*sed1*Δ (*MAT***a**, *his3*Δ1, *leu2*Δ0, *met15*Δ0, *ura3*Δ0, *YDR077w::KanMX4*) (EUROSCARF, Frankfurt, Germany) was used for displaying HA. *E. coli* transformants were grown at 37 °C in Luria–Bertani medium (1 % [w/v] tryptone, 0.5 % [w/v] yeast extract, and 1 % [w/v] sodium chloride) containing 50 μg/mL ampicillin. Yeast transformants were selected on a synthetic dextrose (SDC) solid medium (0.67 % [w/v] yeast nitrogen base without amino acids, 2 % [w/v] glucose, 1 % [w/v] casamino acids, 0.002 % [w/v] adenine, 0.002 % [w/v] l-tryptophan, 2 % [w/v] agar). Isolated transformant colonies were aerobically cultivated at 30 °C in a liquid SDC medium buffered with 5 mM 2-morpholinoethanesulfonic acid (MES; pH 6.5).

### Vectors for cell surface display of human HA

The primers used in this study are listed in Additional file 1: Table S1. The human HA gene was derived from the Influenza A H1N1 (A/New Caledonia/20/99) HA ORF mammalian expression plasmid (Sino Biological, Beijing, China). The DNA fragments encoding a binding domain (human short HA, amino acid residues No. 36–268) or encoding a binding domain and stem region (human long HA, amino acid residues No. 14–515) were PCR-amplified from the plasmid using the appropriate primers (“human short HA Forward” and “human short HA Reverse” for human short HA, or “human long HA Forward” and “human long HA Reverse” for human long HA). The DNA fragments were inserted into *Bgl*II/*Xho*I-digested pULD1 (Kuroda et al. 2009), which encodes the C-terminal half of α-agglutinin downstream of the multiple cloning sites, using an In-Fusion HD Cloning kit (Clontech, Mountain view, CA). The resultant plasmids were named pULD-hSHA^α2,6^ and pULD-hLHA^α2,6^. The plasmids used for displaying the head domain recognizing α2,3-linked sialic acid side chains on surface glycoproteins were constructed by mutating pULD-hSHA^α2,6^ or pULD-hLHA^α2,6^ using a QuikChange Site-Directed Mutagenesis kit (Agilent Technologies, Santa Clara, CA) and the “N190E Forward” and “N190E Reverse” or “D225G Forward” and “D225G Reverse” primers. The resultant plasmids were named pULD-hSHA^α2,6*^ and pULD-hLHA^α2,6*^, respectively. pULD1-strep displaying a strep-tag instead of a FLAG-tag, was used as a negative control for immunofluorescence staining (Kuroda et al. 2009).

### Transformation of yeast

Yeasts were transformed using the Frozen-EZ Yeast Transformation-II kit (Zymo Research, Irvine, CA). After the introduction of plasmids, the yeast transformants were selected on a uracil-deficient SDC solid medium.

### Collection and preparation of red blood cells

Rabbit, chicken, horse (Nippon Biotest Laboratories, Tokyo, Japan), and swine (Funakoshi, Tokyo, Japan) erythrocytes were used for the HA assay. Aliquots (4 mL) of erythrocytes were washed three times with phosphate-buffered saline (PBS; pH 7.4) by centrifugation at 100×*g* for 10 min, and were then resuspended in PBS (pH 7.4) at final working solution (1.0 %).

### Hemagglutination assay

Yeast transformants were precultivated in buffered SDC medium (pH 6.5) for 24 h and the main cultivation was initiated at an initial optical density (OD_600_) of 0.1 in 10 mL buffered SDC medium (pH 6.5). After 72 h cultivation at 30 °C, the cells were harvested and resuspended to 2.0 × 10^8^ cells/mL. Cell suspensions (50 µL) were diluted 2-, 4-, and 8-fold with PBS (pH 7.4) in a 96-well microplate (FALCON 353910, CA). The yeast cells were mixed with 50 μL erythrocyte suspension (1.0 %) and incubated at 25 °C for 2 h. Active HA yields the “cross-linked structure”, while HA lacking binding ability forms “dot-like aggregation” in the center of the well (see Fig. 2). To investigate the relative binding abilities of HA, a quantitative analysis of dot-like aggregation in the well was performed using ImageJ software (National Institutes of Health, MD) (Lazic 2009). Images were analyzed after background correction using the rolling ball radius tool (50 pixels). The area of “dot-like aggregation” of erythrocytes within each negative control well (OD_600_ = 10) was defined as the measurement area, which was then used to evaluate erythrocyte levels as raw integrated density values (sum of pixel values) in other wells (OD_600_ = 10) (Lazic 2009). The results of this analysis were shown as the ratio to negative controls from each of six separate images (Additional file 1: Figure S1). Differences found to be significant by the Steel–Dwass test (*P* < 0.01) were considered to be indicative of HA binding ability.

### Immunofluorescent staining

A total of 3.0 × 10^7^ yeast cells were washed once with 1 mL PBS (pH 7.4) and then resuspended in PBS (pH 7.4) containing 1.0 % (w/v) bovine serum albumin. The yeast cells were incubated at room temperature for 30 min with rotary shaking (25 rpm; RT-50, TAITEC, Saitama, Japan). The yeast cells were incubated with an anti-FLAG M2 mouse monoclonal antibody (Sigma–Aldrich, MO) at a 1:300 dilution for 1.5 h at room temperature. The yeast cells were washed with PBS (pH 7.4) and mixed with Alexa Flour 488-conjugated anti-mouse IgG antibody (Invitrogen, Carlsbad, CA) at a 1:300 dilution for 1 h at room temperature. After washing twice with PBS (pH 7.4), the yeast cells were resuspended to 10^8^ cells/mL by PBS (pH 7.4) for fluorescence observation. Fluorescence was detected using an inverted microscope (Olympus IX71, Tokyo, Japan) through a U-MNIBA2 mirror unit with a BP470–490 excitation filter, DM505 dichroic mirror, and a BA510–550 emission filter (Olympus). Live images were obtained using Aqua-Cosmos 2.0 software (Hamamatsu Photonics, Shizuoka, Japan) controlling a digital charge-coupled device camera (C4742-95-12ER, Hamamatsu Photonics). To determine the display efficiency, the fluorescence intensities were measured by using a Fluoroskan Ascent Fluorometer (Labsystems, Helsinki, Finland) with excitation and emission wavelengths of 485 and 527 nm, respectively. A total of 2.0 × 10^7^ yeast cells were used for measurement.

## Results

### Display of human HAs on the yeast cell surface

**Fig. 1:**
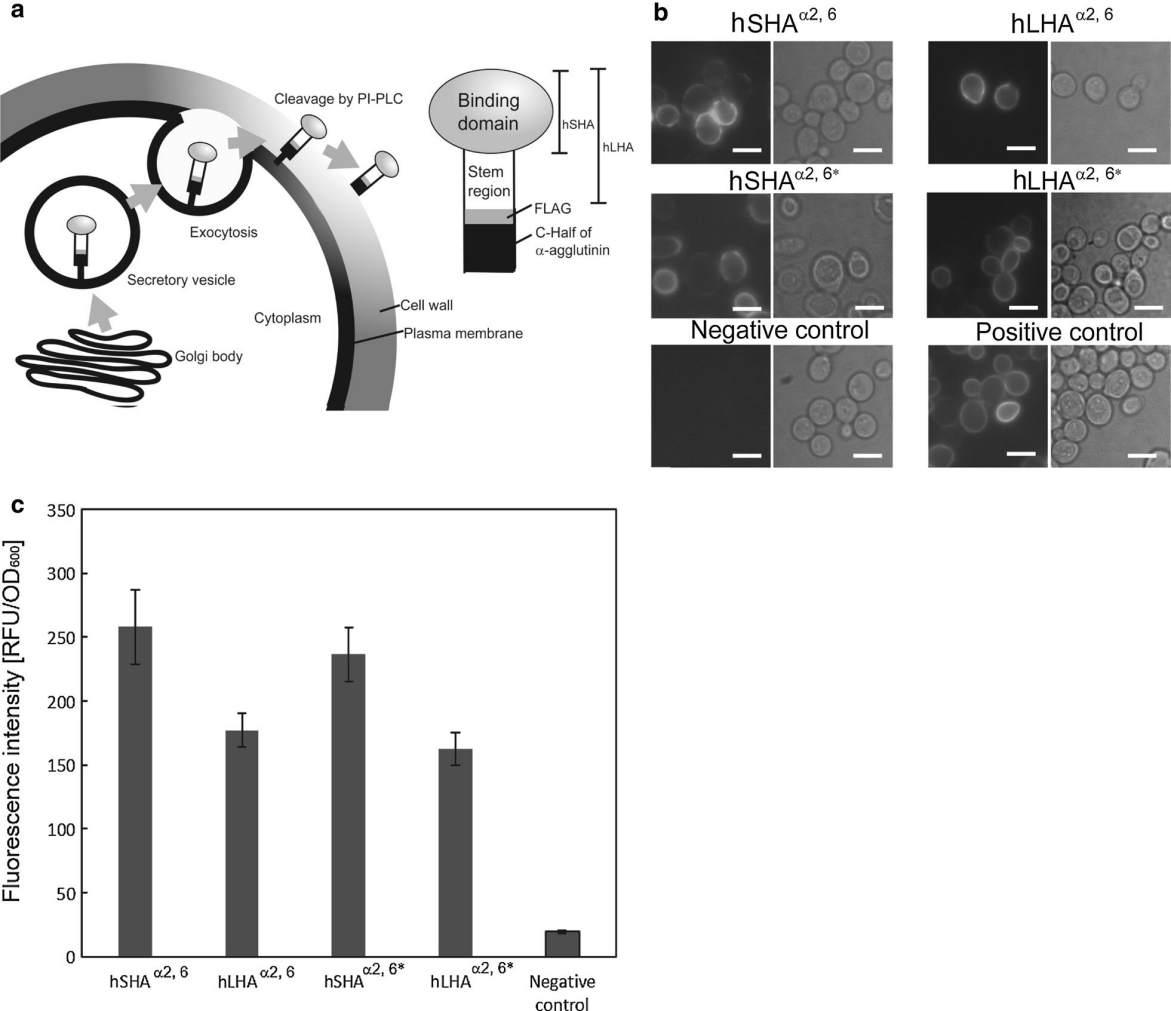
Display of HAs on yeast cell surfaces. **a** Scheme of yeast cell surface display of hHAs^α2,6^ and hHAs^α2,6*^. *PI-PLC* indicates phosphatidylinositol-specific phospholipase C. hHAs^α2,6*^ (hSHA^α2,6*^ and hLHA^α2,6*^) represent the hHAs^α2,6^ with two point mutations (N190E and D225G). **b** Immunofluorescence staining of the engineered yeast cells. *Scale bars*: 5 μm. **c** Comparison of the display efficiencies as relative fluorescence intensities following the immunofluorescence staining. *Values* represent mean ± standard error of the mean (SEM) based on three independent experiments

The HA0 protein of influenza viruses reportedly needs to be cleaved by host proteases to yield two subunits, HA1 and HA2 (Skehel and Wiley 2000; Stieneke-Grober et al. 1992). In the current study, two kinds of yeasts displaying human short HA (hSHA^α2,6^) and human long HA (hLHA^α2,6^) were constructed (Additional file 1: Figure S2). hSHA^α2,6^ contains only the α2,6-linked sialic acid binding site (HA1), while hLHA^α2,6^ contains both the sialic acid binding site and the stem domain (HA1 and HA2) (Fig. 1a). Fluorescent signals due to hHAs^α2,6^ (hSHA^α2,6^ and hLHA^α2,6^) were detected on the yeast cell surface following immunofluorescence staining (Fig. 1b); however, the fluorescence intensity from yeast cells displaying hLHA^α2,6^ was lower than that of yeast cells displaying hSHA^α2,6^ (Fig. 1c). These results indicate that both hHAs^α2,6^ (hSHA^α2,6^ and hLHA^α2,6^) were correctly displayed, although hSHA^α2,6^ displayed more efficiently than hLHA^α2,6^.

### Binding abilities of yeast-displayed human HAs

**Fig. 2:**
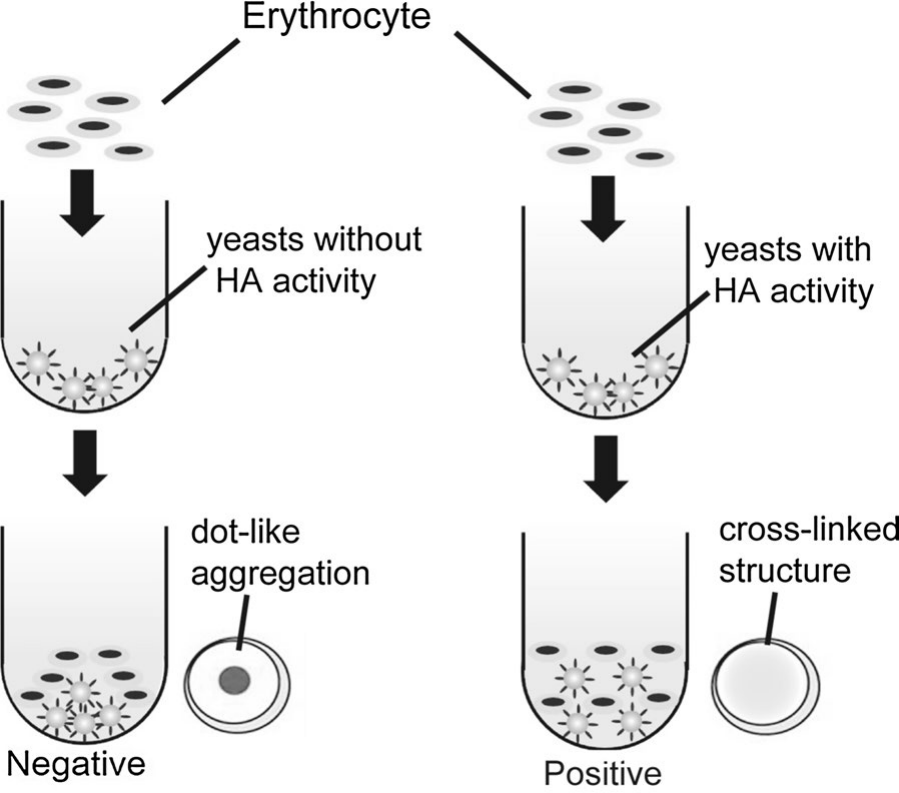
Scheme of hemagglutination assay. “Cross-linked structures” and “dot-like aggregation” are illustrated

**Fig. 3:**
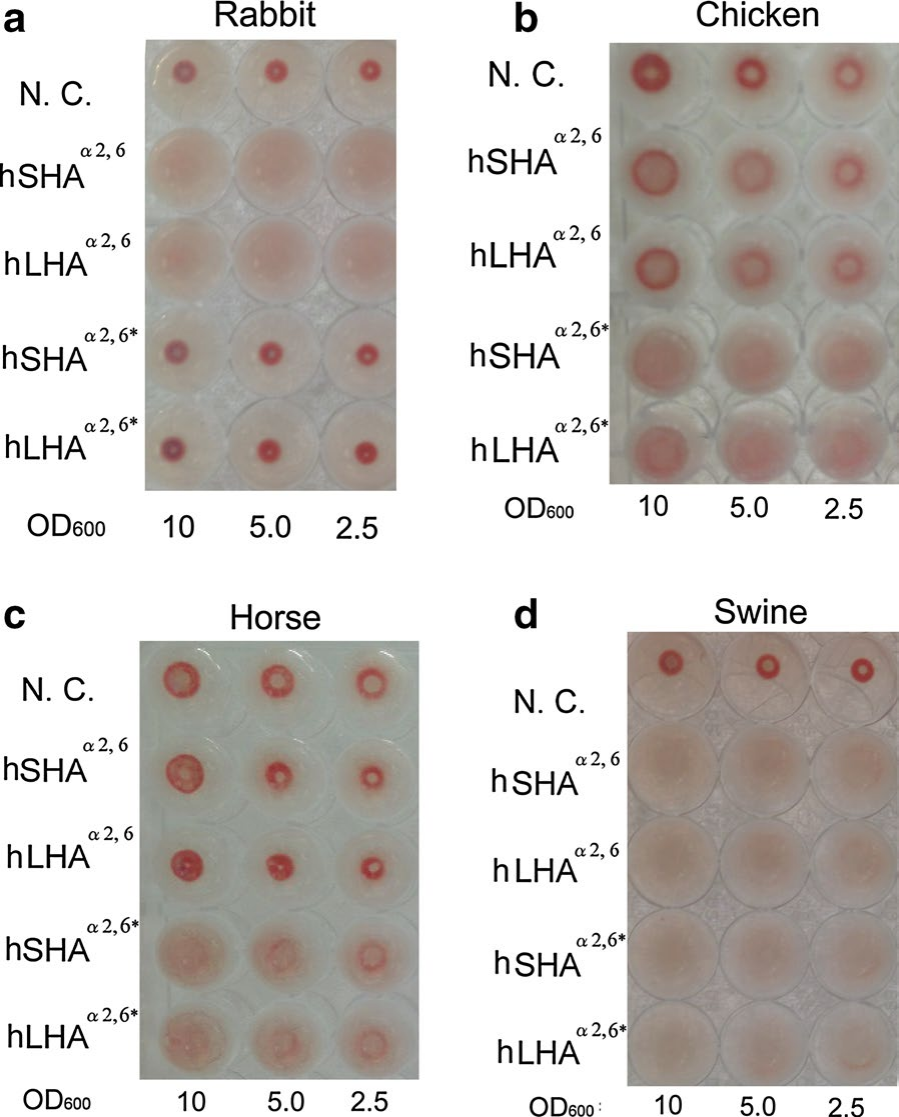
Hemagglutination assay results. **a** Rabbit erythrocytes, **b** chicken erythrocytes, **c** horse erythrocytes, and **d** swine erythrocytes. Yeast cells were diluted from 10 to 2.5 of OD_600_

The binding abilities of the human HAs displayed by yeast cells were confirmed by the hemagglutination assay described in “Materials and methods”. When active HA binds to sialic acid side chains on surface glycoproteins of erythrocytes, a “cross-linked structure” is formed, appearing as a uniform reddish color in the culture plate well. In the case of HA lacking these binding capabilities, on the other hand, the cross-linked structure is not formed, and “dot-like aggregation” is formed in the center of the wells instead (Fig. 2). Rabbit erythrocytes, which have α2,6-linked sialic acid side chains on their surface glycoproteins (Tumpey et al. 2007), were initially used in this study. The hSHA^α2,6^ and hLHA^α2,6^ displayed on yeast cell surface were found to form cross-linkages with the rabbit erythrocyte cells, while the yeast cells displaying only the FLAG-tag (negative control) did not form such cross-linkages (Fig. 3a). To evaluate the relative binding abilities of HA, the sum of pixel values within each well was measured as described in “Materials and methods”. Significant differences were measured between the negative control and hSHA^α2,6^ as well as between the negative control and hLHA^α2,6^ (Additional file 1: Figure S1). These results indicate that the HA-displaying yeasts recognized α2,6-linked sialic acid side chains on surface glycoproteins of rabbit erythrocytes.

### Binding preferences of mutated human HAs on yeast cell surfaces

To further characterize HA on the yeast cell surface, we investigated whether the altered binding site affected the recognition properties of the displayed HA. On the virus surface, HA distinguishes between α2,3- and α2,6-linked sialic acid side chains (Ito et al. 1997). In general, influenza viruses isolated from humans bind to α2,6- but not α2,3-linked sialic acid side chains (Gagneux et al. 2003); however, the 190E and 225G mutations in HAs derived from the human H1 subtype reportedly yielded a change in recognition from α2,6- to α2,3-linked sialic acid side chains (Tumpey et al. 2007). Recognition change can be confirmed using erythrocytes from different hosts (Ito et al. 1997). Chicken erythrocytes, for example, contain more α2,3- than α2,6-linked sialic acid side chains, while rabbit erythrocytes contain mainly α2,6-linked sialic acid side chains (Tumpey et al. 2007). We investigated in this study whether the introduction of two point mutations (N190E and D225G) into hHAs^α2,6^ altered the recognition from α2,6- to α2,3-sialic acid side chains. Human-based short HA (hSHA^α2,6*^) and human-based long HA (hLHA^α2,6*^) were constructed by introducing the two point mutations (N190E and D225G) into hSHA^α2,6^ and hLHA^α2,6^ (Additional file 1: Figure S2). Display of hSHA^α2,6*^ and hLHA^α2,6*^ on yeast cells was confirmed by immunofluorescence (Fig. 1b, c). The yeast-displayed hSHA^α2,6*^ and hLHA^α2,6*^ exhibited binding capabilities for α2,3-linked sialic acid side chains on surface glycoproteins of erythrocyte derived from chicken, while hHAs^α2,6^ did not (Fig. 3b; Additional file 1: Figure S1). Therefore, the two point mutations were shown to alter recognition from α2,6- to α2,3-linked sialic acid side chains, suggesting that the HAs on yeast cell surfaces exhibited similar binding characteristics to those of native HAs on the virus.

The binding preferences of hHAs^α2,6^ and hHAs^α2,6*^ (hSHA^α2,6*^ and hLHA^α2,6*^) toward horse and swine erythrocytes were investigated. Horse erythrocytes contain mainly α2,3-linked sialic acid side chains on surface glycoproteins and swine erythrocytes contain both α2,3- and α2,6-linked sialic acid side chains on their surface glycoproteins (Ito et al. 1997). hHAs^α2,6*^ was shown to form cross-linkages with the horse erythrocytes (Fig. 3c; Additional file 1: Figure S1) and in the case of the swine erythrocytes, both hHAs^α2,6^ and hHAs^α2,6*^ formed cross-linkages (Fig. 3d; Additional file 1: Figure S1). Overall, hHAs^α2,6^ were therefore shown to recognize rabbit and swine erythrocytes, while hHAs^α2,6*^ was shown to recognize chicken, horse, and swine erythrocytes. These recognition specificities are consistent with the distribution of sialic acid side chains on the respective surface glycoproteins (Ito et al. 1997; Medeiros et al. 2001).

## Discussion

In this study, the binding sites of hHAs^α2,6^ and hHAs^α2, 6*^ were successfully displayed on yeast cell surface (Fig. 1b). The binding abilities of the displayed hHAs^α2,6^ were evaluated by hemagglutination assay using rabbit erythrocytes whose surface glycoproteins contain α2,6-linked sialic acid side chains (Fig. 3a; Additional file 1: Figure S1). Altered recognition of HA to α2,3-linked sialic acid side chains was achieved by mutagenesis (Fig. 3b: Additional file 1: Figure S1). The displayed hHAs^α2, 6*^ recognized α2,3-linked sialic acid side chains of chicken erythrocyte surface glycoproteins. The relative binding abilities of hHAs^α2,6^ and hHAs^α2,6*^ toward erythrocytes derived from various species were assessed, and differences between HAs and negative controls, significant by the Steel–Dwass test (*P* < 0.01), were considered indicative of binding capacity of the HA in question (Additional file 1: Figure S1). Chicken erythrocytes contain mainly α2,3- and α2,6-linked sialic acid side chains of surface glycoproteins (Medeiros et al. 2001). In this study, hHAs^α2,6^ did not form cross-linkages with chicken erythrocytes (Fig. 3b; Additional file 1: Figure S1) and the number of α2,6-linked sialic acid side chains on the chicken erythrocytes was thus considered insufficient for cross-linkages to form (Table 1). The hHAs^α2,6*^ formed cross-linkages with horse erythrocytes (Fig. 3c; Additional file 1: Figure S1) and both the hHAs^α2,6^ and the hHAs^α2,6*^ formed cross-linkages with swine erythrocytes (Fig. 3d; Additional file 1: Figure S1). These findings suggest that the two amino acid substitutions ((N190E and D225G) in the HAs on the yeast cell surfaces altered the HA binding preferences, which is in agreement with previously reported findings (Ito et al. 1998). These results demonstrate that the yeast cell surface display system described here is a promising strategy for preparing functional HA mutants.

**Table 1:** Hemagglutination assay with erythrocytes from different animal species

	Hemagglutination with erythrocytes from			
	Rabbit	Chicken	Horse	Swine
Relative abundance^a^	α2, 6	α2, 6 << α2, 3	α2, 3	α2, 6 = α2, 3
Binding activity of HAs^α2,6^	+	−	−	+
Binding activity of HAs^α2,6*^	−	+	+	+

“+” indicates positive binding abilities and “−” indicates no abilities

^a^Distribution of sialic acid-side chains on surface glycoproteins of the erythrocytes from animal species (Ito et al. 1997; Medeiros et al. 2001)

In the yeast cell surface engineering reported here, both hSHA^α2,6^ and hLHA^α2,6^ were successfully displayed on the yeast cell surface (Fig. 1b). Both hSHA^α2,6^ and hLHA^α2,6^ were shown to have similar binding capabilities (Fig. 3a; Additional file 1: Figure S1), suggesting that the yeast-displayed hLHA^α2,6^ did not require protease-mediated cleavage for its binding capabilities (Stevens et al. 2006). Glycan arrays have been used to investigate the binding abilities of HA (Stevens et al. 2006; Kumari et al. 2007). For such glycan arrays, HA cleavage by protease treatments was not required for assessments of the binding abilities of HA (Stevens et al.  2006; Kumari et al. 2007). Similarly, HAs displayed on yeast cell surfaces exhibited binding abilities without HA processing by exogenous proteases. Unlike glycan arrays (Stevens et al. 2006; Kumari et al. 2007), yeast cell surface engineering does not require time-consuming purification and immobilization steps. In glycan arrays, HA must be directly or indirectly labelled with a fluorescent tag to allow for binding events to be detected (Stevens et al. 2006). Because of the weak affinity of HA for sialic acids of proteins, glycan arrays sometimes fail to detect binding events (Stevens et al. 2006; Sauter et al. 1989). The display of HA on yeast cell surfaces, on the other hand, may represent a convenient approach by which HA binding specificities can be confirmed.

Recombinant baculoviruses are widely used to express HA genes in cultured insect cells (Wang et al. 2006; Treanor et al. 2013); however, HA production using this approach may take more than a month (Wang et al. 2006; Treanor et al. 2013). With the yeast display method, on the other hand, HA can be prepared within several days and does not require purification.

Human HA consisting of only the binding site (hSHA) was displayed approximately 1.5 times more effectively than human HA consisting of both the binding site and the stem region (hLHA) (Fig. 1b, c). The molecular mass of displayed proteins on the yeast cell surface affects fluorescence intensities of cells after immunofluorescent staining (Nishitani et al. 2010; Shibasaki et al. 2001). Both hSHA and hLHA exhibited binding abilities; however, hLHA-displaying yeasts would be more useful in inhibitor screening assays since it is more likely that inhibitors would bind not only to the binding site but also to the stem region.

In conclusions, a yeast cell surface system reported on here allows for direct measurement of the binding abilities of HAs without protein purification steps and thus would allow for the preparation of an HA mutant library as well as an NA mutant library as previously reported (Shigemori et al. 2013). This application would be advantageous in the analysis of newly emerging influenza virus mutants, thereby contributing to the prompt development of antiviral drugs. This yeast cell surface display system would therefore contribute to the prevention of influenza virus pandemics caused by frequent viral mutations.

## Additional file

10.1186/s13568-016-0179-y List of primers used in this study (Table S1). Box plots showing the ratio of sum of pixel values for (A) rabbit, (B) chicken, (C) horse, and (D) swine erythrocytes (Fig. S1). Sequence alignment of hHAs^α2,6^ and hHAs^α2,6*^ (Fig. S2).
