# Supplementary material for: Rapid preparation of mutated influenza hemagglutinins for influenza virus pandemic prevention
Source: AMB Express. 2016 Jan 21;6:8. doi: 10.1186/s13568-016-0179-y (PMC4722048; doi:10.1186/s13568-016-0179-y)
Supplement: Supplementary file 1 — 10.1186/s13568-016-0179-y List of primers used in this study (Table S1). Box plots showing the ratio of sum of pixel values for (A) rabbit, (B) chicken, (C) horse, and (D) swine erythrocytes (Fig. S1). Sequence alignment of hHAsα2,6 and hHAsα2,6* (Fig. S2). [file 13568_2016_179_MOESM1_ESM.pdf]

| | |
|---|---|
| article title | Rapid preparation of mutated influenza hemagglutinins for influenza virus pandemic prevention |
| journal name | AMB Express |
| author names | Ryosuke Nishioka, Atsushi Satomura, Junki Yamada, Kouichi Kuroda, Mitsuyoshi Ueda |
| affiliation and e-mail address of the corresponding author | Corresponding auther : Mitsuyoshi Ueda<br>Division of Applied Life Sciences, Graduate School of Agriculture, Kyoto University, Sakyo-ku, Kyoto 606-8502, Japan |
| | miueda@kais.kyoto-u.ac.jp |

**Table S1 List of primers**

| Name | Sequence (5’ to 3’) |
|---|---|
| human short HA Forward | TTGCTCGTTTCTGCCCTGTGTCTGCTGAAAGGCATTGC |
| human short HA Reverse | GTAATCAGATCCACCTGTCTGACACTTGGCATCACACTC |
| human long HA Forward | TTGCTCGTTTCTGCCAACAACAGCACAGACACAGTGGACAC |
| human long HA Reverse | GTAATCAGATCCACCCTGGTAGACTCCCATACTCTCCAATTTCAC |
| N190E Forward | GAGTGCATCACCCTCCAAACATTGGA<u>GAA</u>CAGAGGGCTCTCTACCACACAGAGAATGCC |
| N190E Reverse | GGCATTCTCTGTGTGGTAGAGAGCCCTCTG<u>TTC</u>TCCAATGTTTGGAGGGTGATGCACTC |
| D225G Forward | CCTGAGATTGCCAAGAGACCAAAGGTGAGG<u>GGT</u>CAGGAGGGCAGGATAAACTACTACTGGACC |
| D225G Reverse | GGTCCAGTAGTAGTTTATCCTGCCCTCCTG<u>ACC</u>CCTCACCTTTGGTCTCTTGGCAATCTCAGG |

The underlines indicate mutation point.

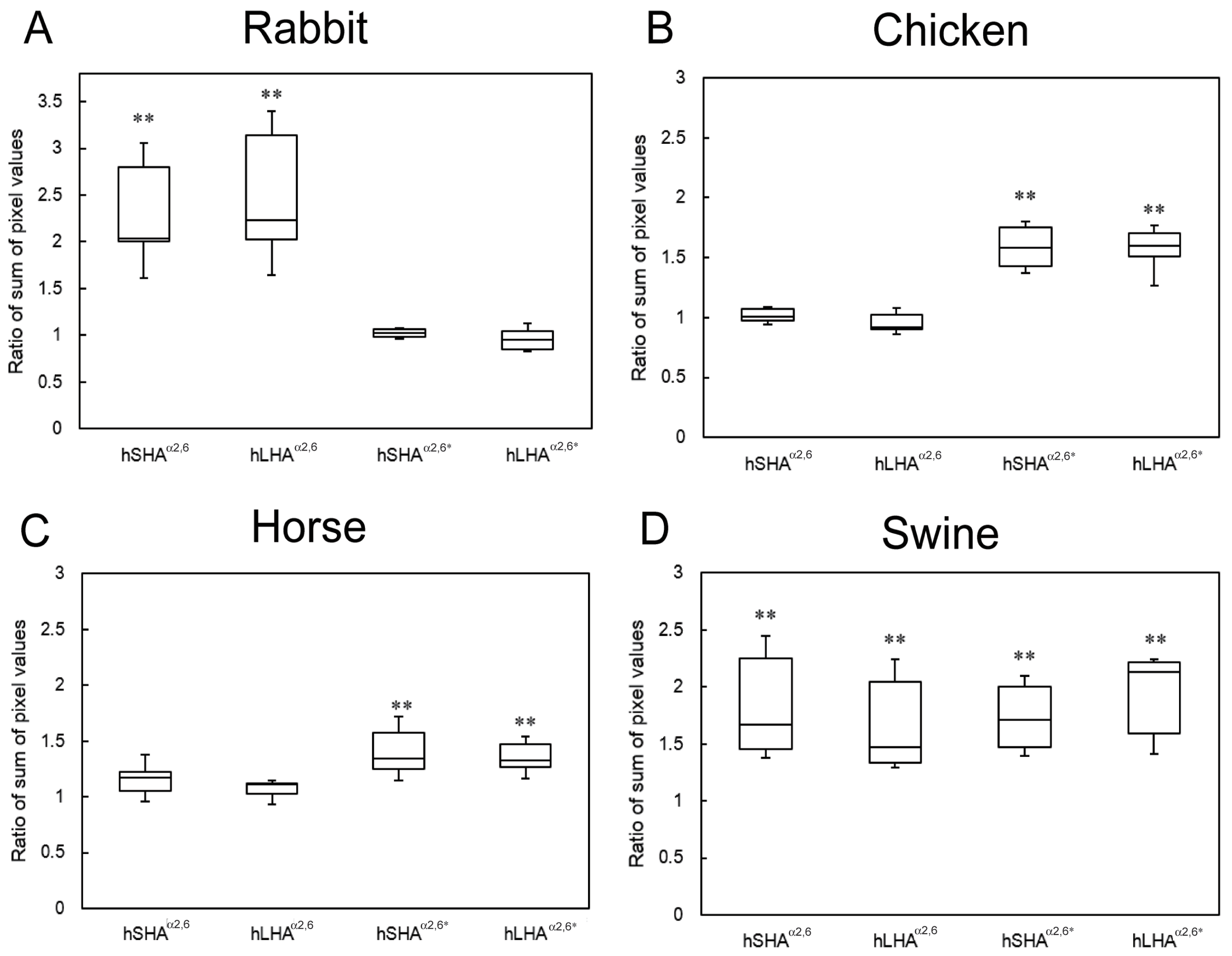


**Supplemental Figure S1 Box plots showing the ratio of sum of pixel values for (A) rabbit, (B) chicken, (C) horse, and (D) swine erythrocytes.**

hSHA$^{\alpha 2,6*}$ and hLHA$^{\alpha 2,6*}$ represent the hSHA$^{\alpha 2,6}$ and hLHA$^{\alpha 2,6}$ species with two point mutations each (N190E and D225G). In each box plot, the y-axis represents as a relative ratio to negative controls. The center bar shows the median value, the box denotes the 25 and 75% centiles, the upper and lower bars represent the range, and the whiskers indicate the 5 and 95% quantiles. *P*-values were determined by the Steel-Dwass test (*$P < 0.05$, **$P < 0.01$).

```
hSHAα2,6     ---------------------------------CLLKGIAPLQLGNCSVAGWILGNPECEL
hLHAα2,6     NNSTDTVDTVLEKNVTVTHSVNLLEDSHNGKLCLLKGIAPLQLGNCSVAGWILGNPECEL
hSHAα2,6*    ---------------------------------CLLKGIAPLQLGNCSVAGWILGNPECEL
hLHAα2,6*    NNSTDTVDTVLEKNVTVTHSVNLLEDSHNGKLCLLKGIAPLQLGNCSVAGWILGNPECEL
                                       HA1

hSHAα2,6     LISKESWSYIVETPNPENGTCYPGYFADYEELREQLSSVSSFERFEIFPKESSWPNHTVT
hLHAα2,6     LISKESWSYIVETPNPENGTCYPGYFADYEELREQLSSVSSFERFEIFPKESSWPNHTVT
hSHAα2,6*    LISKESWSYIVETPNPENGTCYPGYFADYEELREQLSSVSSFERFEIFPKESSWPNHTVT
hLHAα2,6*    LISKESWSYIVETPNPENGTCYPGYFADYEELREQLSSVSSFERFEIFPKESSWPNHTVT
                                       HA1
                                                                   190
hSHAα2,6     GVSASCSHNGKSSFYRNLLWLTGKNGLYPNLSKSYVNNKEKEVLVLWGVHHPPNIGNQRA
hLHAα2,6     GVSASCSHNGKSSFYRNLLWLTGKNGLYPNLSKSYVNNKEKEVLVLWGVHHPPNIGNQRA
hSHAα2,6*    GVSASCSHNGKSSFYRNLLWLTGKNGLYPNLSKSYVNNKEKEVLVLWGVHHPPNIGEQRA
hLHAα2,6*    GVSASCSHNGKSSFYRNLLWLTGKNGLYPNLSKSYVNNKEKEVLVLWGVHHPPNIGEQRA
                                       HA1
                                 225
hSHAα2,6     LYHTENAYVSVVSSHYSRRFTPEIAKRPKVRDQEGRINYYWTLLEPGDTIIFEANGNLIA
hLHAα2,6     LYHTENAYVSVVSSHYSRRFTPEIAKRPKVRDQEGRINYYWTLLEPGDTIIFEANGNLIA
hSHAα2,6*    LYHTENAYVSVVSSHYSRRFTPEIAKRPKVRGQEGRINYYWTLLEPGDTIIFEANGNLIA
hLHAα2,6*    LYHTENAYVSVVSSHYSRRFTPEIAKRPKVRGQEGRINYYWTLLEPGDTIIFEANGNLIA
                                       HA1

hSHAα2,6     PWYAFALSRGFGSGIITSNAPMDEC-----------------------------------
hLHAα2,6     PWYAFALSRGFGSGIITSNAPMDECDAKCQTPQGAINSSLPFQNVHPVTIGECPKYVRSA
hSHAα2,6*    PWYAFALSRGFGSGIITSNAPMDEC-----------------------------------
hLHAα2,6*    PWYAFALSRGFGSGIITSNAPMDECDAKCQTPQGAINSSLPFQNVHPVTIGECPKYVRSA
                                       HA1

hSHAα2,6     ------------------------------------------------------------
hLHAα2,6     KLRMVTGLRNIPSIQSRGLFGAIAGFIEGGWTGMVDGWYGYHHQNEQGSGYAADQKSTQN
hSHAα2,6*    ------------------------------------------------------------
hLHAα2,6*    KLRMVTGLRNIPSIQSRGLFGAIAGFIEGGWTGMVDGWYGYHHQNEQGSGYAADQKSTQN
                        HA1                    HA2

hSHAα2,6     ------------------------------------------------------------
hLHAα2,6     AINGITNKVNSVIEKMNTQFTAVGKEFNKLERRMENLNKKVDDGFLDIWTYNAELLVLLE
hSHAα2,6*    ------------------------------------------------------------
hLHAα2,6*    AINGITNKVNSVIEKMNTQFTAVGKEFNKLERRMENLNKKVDDGFLDIWTYNAELLVLLE
                                                   HA2

hSHAα2,6     ------------------------------------------------------------
hLHAα2,6     NERTLDFHDSNVKNLYEKVKSQLKNNAKEIGNGCFEFYHKCNNECMESVKNGTYDYPKYS
hSHAα2,6*    ------------------------------------------------------------
hLHAα2,6*    NERTLDFHDSNVKNLYEKVKSQLKNNAKEIGNGCFEFYHKCNNECMESVKNGTYDYPKYS
                                                   HA2

hSHAα2,6     ----------------------
hLHAα2,6     EESKLNREKIDGVKLESMGVYQ
hSHAα2,6*    ----------------------
hLHAα2,6*    EESKLNREKIDGVKLESMGVYQ
                        HA2
```

**Supplemental Figure S2 Sequence alignment of hHAs$^{\alpha2,6}$ and hHAs$^{\alpha2,6*}$.**

The full-length sequences of hSHA$^{\alpha2,6}$, hLHA$^{\alpha2,6}$, hSHA$^{\alpha2,6*}$, and hLHA$^{\alpha2,6*}$ are shown. Multiple sequence alignments were generated using the ClustalW program (http://www.ebi.ac.uk/Tools/msa/clustalw2/). The underlined sequence labelled HA1 represents the head region that binds to sialic acid side chains on surface glycoproteins (Skehel and Wiley 2000), while the underlined sequence labelled HA2 represents the stem region required for membrane fusion activity and infectivity (Skehel and Wiley 2000). The shaded amino acids show the amino acids mutated to generate hHAs$^{\alpha2,6*}$.
